# Functional and Structural Analysis of C-Terminal BRCA1 Missense Variants

**DOI:** 10.1371/journal.pone.0061302

**Published:** 2013-04-17

**Authors:** Francisco Quiles, Juana Fernández-Rodríguez, Roberto Mosca, Lídia Feliubadaló, Eva Tornero, Joan Brunet, Ignacio Blanco, Gabriel Capellá, Miquel Àngel Pujana, Patrick Aloy, Alvaro Monteiro, Conxi Lázaro

**Affiliations:** 1 Hereditary Cancer Program, Catalan Institute of Oncology-(Bellvitge Institute for Biomedical Research; Girona Institute for Biomedical Research; Germans Trial i Pujol Research Institute) (ICO-IDIBELL, ICO-IdIBGi, ICO-IGTP), L'Hospitalet de Llobregat, Barcelona, Spain; 2 Institute for Research in Biomedicine (IRB) Barcelona, Joint IRB-BSC Program in Computational Biology, Barcelona, Spain; 3 Breast Cancer Unit, Translational Research Laboratory, Catalan Institute of Oncology (ICO), Bellvitge Institute for Biomedical Research (IDIBELL), L'Hospitalet de Llobregat, Barcelona, Spain; 4 Institució Catalana de Recerca i Estudis Avançats (ICREA), Barcelona, Spain; 5 Cancer Epidemiology Program, H. Lee Moffitt Cancer Center & Research Institute, Tampa, Florida, United States of America; IFOM, Fondazione Istituto FIRC di Oncologia Molecolare, Italy

## Abstract

Germline inactivating mutations in *BRCA1* and *BRCA2* genes are responsible for Hereditary Breast and Ovarian Cancer Syndrome (HBOCS). Genetic testing of these genes is available, although approximately 15% of tests identify variants of uncertain significance (VUS). Classification of these variants into pathogenic or non-pathogenic type is an important challenge in genetic diagnosis and counseling. The aim of the present study is to functionally assess a set of 7 missense VUS (Q1409L, S1473P, E1586G, R1589H, Y1703S, W1718L and G1770V) located in the C-terminal region of BRCA1 by combining *in silico* prediction tools and structural analysis with a transcription activation (TA) assay. The *in silico* prediction programs gave discrepant results making its interpretation difficult. Structural analysis of the three variants located in the BRCT domains (Y1703S, W1718L and G1770V) reveals significant alterations of BRCT structure. The TA assay shows that variants Y1703S, W1718L and G1770V dramatically compromise the transcriptional activity of BRCA1, while variants Q1409L, S1473P, E1586G and R1589H behave like wild-type BRCA1. In conclusion, our results suggest that variants Y1703S, W1718L and G1770V can be classified as likely pathogenic *BRCA1* mutations.

## Introduction

Between 10 and 20% of the breast cancer cases appearing in the general population present familial history of the disease [1]. Mutations in *BRCA1* and *BRCA2* genes confer high lifetime risks of breast and ovarian cancer, among other neoplasias [2], [3]. Inactivating germline mutations in these genes account for 20–50% of familial cases, depending on the population [4]. Thus, genetic analysis of *BRCA1* and *BRCA2* is a cornerstone of genetic counseling practice. However, classification of genetic variants as pathogenic is challenging, particularly for missense changes and for silent or intronic variants that cannot be directly associated with increased cancer risk and are classified as variants of uncertain significance (VUS), which are found in 13% of *BRCA1* and *BRCA2* genetic tests [5]. Diverse multifactorial likelihood algorithms have been developed and applied for both *BRCA1* and *BRCA2* variants (reviewed in Spurdle et al., 2011) [6]. These models use the combination of a number of independent features (sequence conservation, type of amino-acid change, familial co-segregation, family and personal cancer history, tumor data, co-occurrence with a deleterious mutation, and severity of amino acid change) to establish the likelihood that a given VUS is pathogenic or non-pathogenic. In many cases there is not enough data to classify such mutations for clinical purposes. Therefore, functional analyses that assess specific properties of BRCA1 or BRCA2 may help to classify VUS [7]. In particular, *BRCA1* encodes for a protein of 1,863 amino acids and with multiple functional domains [8]. Several functional assays have been conducted to evaluate VUS in BRCA1 at the level of its global and domain-based functions, including ubiquitin ligase activity assays, protease sensitivity assays, phosphopeptide binding assays, small colony phenotype assays, yeast localization phenotype assays, and embryonic stem cell-based functional assays (review in Millot et al., 2012) [9]. In the work presented here, we combine a functional assay - the transcription activation (TA) assay, which is based on the function of the BRCA1 carboxy-terminal region (aa 1396–1863) in transcriptional activation domain when linked to a sequence-specific DNA binding module - [10] with protein structural analyses [11] to assess the functional impact of seven BRCA1 C-terminal VUS. Our results indicate that three of them (Y1703S, W1718L, G1770V) have significant functional impact and may represent pathogenic *BRCA1* variants while the remaining four do not have a functional impact.

## Materials and Methods

### *BRCA*1 VUS

**Figure 1:**
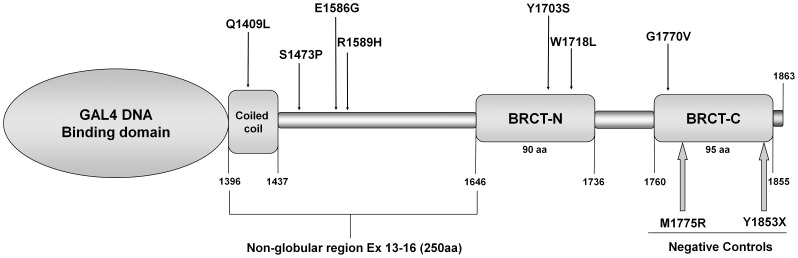
Illustration depicting the location of the C-terminal BRCA missense variants and negative controls. This Figure shows the position of missense variants analyzed in the present study (black arrows) and the negative controls (closed gray arrows, underlined) within the vector used in the TA experiments.

Seven missense BRCA1 C-terminal variants (Q1409L, S1473P, E1586G, R1589H, Y1703S, W1718L and G1770V), identified through genetic testing of patients with suspicion of Hereditary Breast and Ovarian Cancer Syndrome (HBOCS) as part of the Hereditary Cancer Program of the Catalan Institute of Oncology, were included in this study (Figure 1). Written informed consent was obtained from all subjects. The study received the approval of the Ethics Committee of IDIBELL. The pedigrees of these families are depicted in Figure S1. *BRCA1* and *BRCA2* genetic analyses consisted of screening of point mutations and large genomic rearrangements [12]. Genetic tests were carried out once patients had received appropriate genetic counseling and provided written informed consent.

### *In Silico* Predictions

The *BRCA1* sequences containing the VUS were evaluated for their potential pathogenicity using the following algorithms: Align-GVGD [13], PolyPhen-2 [14], SIFT [15], Mutation Assessor [16], PhD-SNP [17], SNAP [18], I-Mutant [19], PON-P [20], and Condel [21]. The Align-GVGD algorithm was used at two depths of alignment (human to frog and human to sea urchin). The results of PolyPhen-2 were retrieved from the original webpage (version 2.2.2) but also from version 2.0.22 run by PON-P and version 1 run by Condel, which use them for weighted average scores.

### Transcription Activation Assay

The assay was based on the mammalian expression of the firefly luciferase gene under the control of five GAL4 binding sites [10]. In this system, pcDNA3 constructs containing the GAL4 DNA binding domain (DBD) fused to the wild-type or VUS-containing BRCA1 C-terminal region were used. Pathogenic mutation controls (M1775R and Y1853X) were included in the assays. Constructs containing the VUS were generated by site-directed mutagenesis using the QuickChange II Site-Directed Mutagenesis Kit (Agilent Technologies, USA) and the wild-type sequence as a template (U14680). Human embryonic kidney (HEK) 293T cells were co-transfected (X-tremeGENE 9 DNA transfection Reagent, Roche Applied Science, Mannheim, Germany) with the pcDNA3 construct encoding either the wild-type or the VUS sequence, the pG5Luc plasmid that contains the luciferase reporter gene, and the phRG-TK plasmid that contains a *Renilla* luciferase gene under the control of a constitutive thymidine kinase promoter, which acts as an internal control. Luciferase was quantified using the Dual-Luciferase Reporter Assay System (Promega, Madison, USA).

### Protein Structural Rationalizations

To predict the impact of the VUS on both the folding and phosphopeptide binding of the BRCT domains a structural analysis was carried out by mapping the position of the variants on the BRCA1 structures collected from the Protein Data Bank [22] (PDB, www.pdb.org) and analyzing them manually with Pymol (http://www.pymol.org). The structures were collected by BLAST-querying the PDB using the BRCA1 sequence (UniProt accession code P38398). Only the VUS mapped in the BRCT tandem domains (Y1703S, W1718L and G1770V) were analyzed using this approach, as there are no structural data for the upstream domain or regions.

## Results

### *In Silico* Predictions

Several algorithms designed to predict deleterious versus neutral missense changes were used (Table 1). Align-GVGD [13], PolyPhen-2 [14], SIFT [15], Mutation Assessor [16], PhD-SNP [17], SNAP [18] and I-Mutant [19] differ in the properties taken into account to generate the prediction and in the method and possible training for decision-making (reviewed in Thursberg, et al., 2010) [23]. PON-P [20] integrates PolyPhen2, SIFT, PhD-SNP, SNAP and I-Mutant using a random forest method, whereas Condel integrates PolyPhen2, SIFT and Mutation Assessor using a weighted average of the normalized scores of the individual methods. Both integration methods claim to outperform individual ones [20], [21]. Table 1 shows the outcomes of all methods, for comparison with high discrepancy between different programs. The two integrative algorithms indicate that variants Q1409L, S1473P, E1586G and R1589H have a benign effect and that G1770V has a damaging effect, whereas they disagree in their predictions for variants Y1703S and W1718L. Furthermore the results of PON-P and Condel for Q1409L and G1770V strikingly diverge from those of Align-GVGD, which classify Q1409L as most likely to interfere with BRCA1 function and G1770V as less likely using the alignment from human to frog. Also SIFT, Mutation Assessor, PhD-SNP, SNAP and I-Mutant show divergent tendencies.

**Table 1:** *In silico* predictions for pathogenicity of the variants presented in this study.

Predictor^1^		Align-GVGD^2^		PolyPhen-2 v2.2.2 (Original)		PolyPhen-2 v2.0.22 (PON-P)		PolyPhen-2 v1 (Condel)		SIFT		Mutation Assessor		PhD-SNP		SNAP		I-Mutant		PON-P^0^			Condel	
DNAvariant	Protein variant	Human to frog	Human to sea urchin	Prob.	Pred.	Prob.	Class	Prob.	Pred.	Prob.	Class	Funct. Impact Score	Funct. Impact	Pred.	Reliability	Pred.	Accur.	ΔΔG	Pred.	Pred.	Class	Accur.	Prob.	Class
c.4226A>T	Q1409L	C65	C15	0.015	benign	0.995	Del.	0.007	benign	0.00	damaging	1.100	low	N	3	N	60	0.22	N	0.14	N	0.84	0.000	N
c.4417T>C	S1473P	C0	C0	0.004	benign	0.960	Del.	0.124	benign	0.01	damaging	0.975	low	N	3	N	85	−2.07	Dest	0.18	N	0.76	0.018	N
c.4757A>G	E1586G	C0	C0	0.003	benign	0.995	Del.	0.124	benign	0.01	damaging	1.040	low	N	8	P	58	−1.81	Dest	0.12	N	0.84	0.014	N
c.4766G>A	R1589H	C0	C0	0.000	benign	0.000	N	0.000	benign	1.00	tolerated	−1.040	neutral	N	8	N	92	−1.52	Dest	0.00	N	0.86	0.000	N
c.5108A>C	Y1703S	C65	C65	0.430	benign	1.000	Del.	0.689	Possib. damaging	0.00	damaging	2.670	medium	P	9	P	58	−1.44	Dest	0.39	UV	0.60	0.746	Del
c.5153G>T	W1718L	C55	C55	0.086	benign	1.000	Del.	0.497	benign	0.00	damaging	3.105	medium	P	4	P	58	−0.81	N	0.57	UV	0.46	0.065	N
c.5309G>T	G1770V	C0	C0	0.093	benign	0.999	Del.	0.964	possib. damaging	0.00	damaging	2.135	medium	P	8	P	78	−1.20	Dest	0.98	P	0.96	0.906	Del

Each predictor yields its own type of results, usually quantitative and categorical. We have chosen the most representative of each.

1 Predictors PON-P and Condel integrate results from other predictors run in their own servers; as PolyPhen-2 versions and their results differ if they are run in the PolyPhen-2 website or the other 2, all of them are shown for comparison. ^2^Classification of variants in different classes according to Align-GVGD algorithm (Align-Grantham Variation Grantham Deviation; http://agvgd.iarc.fr), C65 means “Most likely to interfere with function, C0 means “least likely” (possible classes are: C65> C55>C45>C35>C25>C15>C0).

Abbreviations: Prob.-Probability; Pred.-Prediction; Funct.-Functional; Accur.-Accuracy; ΔΔG–ΔΔG value (kcal/mol); Del-Deleterious; N-Neutral; Dest- Destabilizing; P-Pathogenic; UV-Unknown Variant.

### Transcription Activation Assay

**Figure 2:**
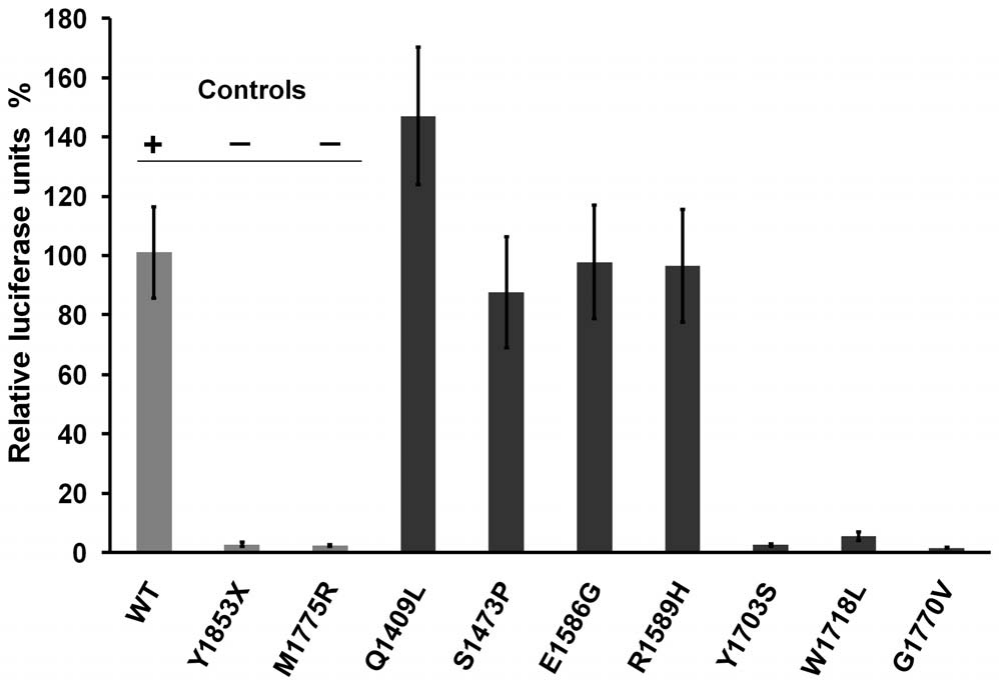
Results of functional assay of the studied missense variants. Percentage of transcriptional activity was expressed as percentage relative to the wild-type construct. Experiments were performed in triplicate and normalized against the *Renilla* luciferase internal control. Results are shown in bars: gray for controls and black for variants.

Using the transcriptional assay, three VUS (Y1703S, W1718L and G1770V) showed a significant decrease in reporter expression compared to wild-type *BRCA1* (Figure 2). The luciferase activity of these three mutants was very similar to that observed for the known pathogenic mutants used as controls: that is <5%, compared to the wild-type construct. Conversely, the remaining four VUS (Q1409L, S1473P, E1586G and R1589H) displayed similar transcriptional activity to that of the wild-type construct.

### Protein Structural Rationalizations

**Figure 3:**
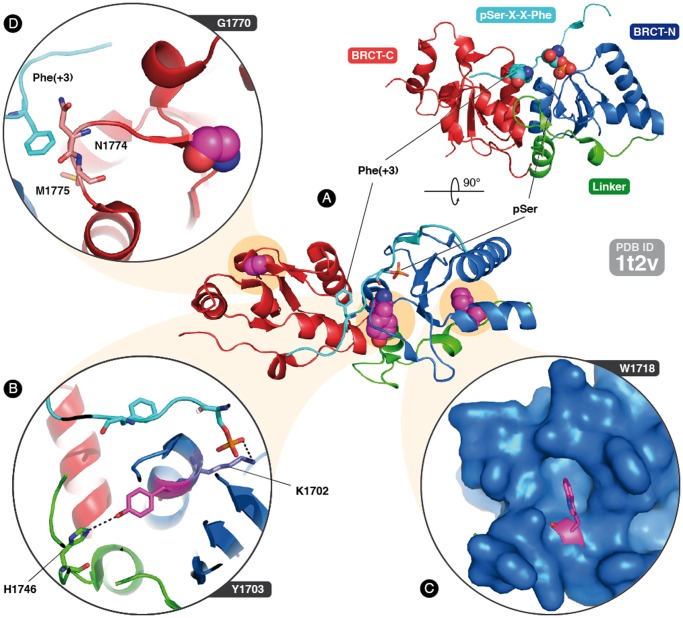
Structural analysis of missense variants lying in the BRCA1 BRCT domain. Representation of the structure of the BRCT tandem repeat from human BRCA1 in complex with a binding phosphopeptide (with the typical binding motif pSer-X-X-Phe, PDB ID 1t2v): in blue, the N-terminal repeat (BRCT-N); in red, the C-terminal repeat (BRCT-C); in green, the linker region between the two; in cyan, the phosphopeptide with the phosphoserine and the key binding residue Phe(+3); in, magenta the three mutated residues. Y1703 (A) forms a hydrogen bond with H1746 and is adjacent to K1702, which binds directly to the pSer residue in the phosphopeptide. W1718 (B) fills the internal core and stabilizes the BRCT-N domain. G1770 (C) gives the loop connecting the β1 strand with α1-helix in the BRCT-C repeat the necessary flexibility to form a tight turn, bringing the residues N1774 and M1775 close to the phosphopeptide for interaction with the Phe(+3) key residue.

A structural analysis was performed for those VUS located in the BRCT domains (Y1703S, W1718L and G1770V). Y1703 is located at the BRCT dual-repeat-interaction interface (Figure 3A), directed towards the linker. It forms a hydrogen bond with H1746 (Figure 3B). The change to a serine, by removal of the aromatic ring, is likely to disrupt this bond and the surrounding hydrophobic interactions and to affect the stability of the domain and the dual-repeat interaction. It may also affect the peptide binding through K1702 (Figure 3B), which interacts directly with the peptide’s phosphoserine. W1718 is loosely packed in the core of the BRCT-N domain and is part of a highly conserved motif within the α3-helix (Figure 3C). The substitution of this bulky residue with a leucine likely destabilizes the folding of the domain, analogously to experimentally confirmed observations of substitution by a cysteine [24], [25]. G1770 is solvent-exposed and located on a tight turn of the loop connecting the β1 strand with the α1 helix in the BRCT-C repeat (Figure 3D). It is highly conserved in the Pfam alignment of the BRCT domain and probably confers to the loop the necessary flexibility for phosphopeptide binding. Substitution by a valine would expose a hydrophobic side chain and confer rigidity to the loop, compromising the binding. In contrast to the other mutations, this change may not affect folding.

## Discussion

This study combined a functional BRCA1 assay and structural analyses to predict whether specific missense variants in the BRCA1 C-terminal region have pathological significance, contributing to breast cancer risk. The variants selected were VUS identified in a clinical scenario, which are located between amino acid residues 1,396 and 1,863 of the full-length BRCA1 sequence and include part of a coiled-coil domain (aa 1,364–1,437) and the BRCT tandem domains (aa 1,646–1,863) (Figure 1). Notably, six of the VUS (Q1409L, S1473P, E1586G, Y1703S, W1718L and G1770V) had not been recorded in the locus-specific database for mutations in *BRCA1* and *BRCA2*, BIC (Breast Cancer Information Core database, http://research.nhgri.nih.gov/bic/) (Figure 1 and Table 1).

The use of different *in silico* prediction algorithms gave contradictory results for some of the variants (Table 1) making it difficult to draw any clear conclusion. For example, there is a striking difference between the results for three versions of PolyPhen-2 as run from different sources; W1718L can yield probabilities of pathogenicity of 0.086, 0.497 and 1.000, while the probabilities for Q1409L, S1473P and E1586G range from values below 0.2 to values above 0.95 in the different versions. If we tried to order the variants according to their deleteriousness as predicted from each of the algorithms, they would yield as many orders as algorithms tested. In general terms, the two integrative programs gave quite similar results, predicting a damaging effect for one of the variants (G1770V) and a benign effect for four of them (Q1409L, S1473P, E1586G and R1589H), while variants Y1703S and W1718L were difficult to classify. Therefore, attending to the results obtained in this study, we consider the use of *in silico* programs not reliable enough to get an approximation of the pathogenicity of a given VUS. In a recent published paper by Li *et al.*, the predictive power of Condel and five individual methods (SIFT and PolyPhen2 among them) in discriminating between pathogenic nsSNVs and other rare nsSNVs, was examined [26]. Their results showed that the predictive power of the combined Condel methodology is not necessarily better than individual methods. Furthermore and in agreement with our data, they show that correlations in the scores between the predictive methods tested are weak or moderate. The observed discrepancies among programs clearly support the need of adding functional assay results in the process of classification of VUS.

The TA assay in mammalian cells was performed as described in previous studies [10]. This assay has been extensively validated, proving to be extremely robust for variants located in the BRCT domain [7], [10], [27], [28]. In a previous study it has been benchmarked using 14 pathogenic and 10 not pathogenic variants, showing high sensitivity and specificity [28]. In this study two constructs containing previously classified pathogenic variants (IARC Class 5), M1775R and Y1853X, were used as negative controls and a construct with the wild-type BRCA1 C-terminal region was used as a positive control. This assay clearly identified a functional defect for three of the variants (Y1703S, W1718L and G1770V), whereas the remaining variants gave results very similar to those of the wild-type controls. It should be noted that residue W1718 appears to be highly prone to mutation, and although the mutation studied here has never been reported, mutations W1718S and W1718C have been described in HBOCS families; in addition, a panel of assays including protease sensitivity, phosphopeptide binding activity and specificity, as well as the TA assay, revealed that both of these variants have strong functional effects [28]. Both constitute large volume changes at a rigid position in a rigid region that is part of the BRCT signature motif WXXXS [29].

Next, to better interpret and understand the functional data obtained, a structural analysis was performed for those VUS located in the BRCT domains. This analysis was limited to the BRCT domain as its structure has been solved [11], making it possible to rationalize whether a given variant alters the domain stability or the phosphopeptide-mediated binding to phosphorylated protein partners (Figure 3). The results indicated that the three studied VUS in the BRCT domains seem to damage proper BRCA1 structure. Interestingly, the experimental data obtained in the TA assay support these structural predictions.

The pedigree structure of patients with these missense mutations was, in general, not very informative for co-segregation analysis (see Figure S1), either due to the lack of relatives with breast (or ovarian) cancer or because no DNA samples for other relatives were available. Nevertheless, it is worth noting that variant Q1409L, which appears to have no effect on TA activity in the present study, was found in a patient with a pathogenic mutation in *BRCA2* (c.5720_5723delCTCT), and although a small number of carriers of two pathogenic mutations - one in *BRCA1* and one in *BRCA2* - have been described worldwide this event has a very low probability. In spite of this, and due to the fact that mutation Q1409L is mapped in the BRCA1/PALB2 interaction region, it would be worth to test if mutant L1409 is hampering the BRCA1/PALB2 interaction [30]. Moreover, mutation G1770V, which shows a clear impairment of TA activity, has been found in two families of Moroccan origin presumed to be independent. In both cases, patients suffer from breast or ovarian cancer at a very early age of onset (27 and 30 years of age, respectively).

In summary, in a clinical context, extensive worldwide analysis of *BRCA1* has identified a large number of VUS, most of them unique, which need to be classified for the purposes of diagnosis and genetic counseling. By combining functional analysis and structural rationalizations, this study shows that three *BRCA1* VUS (Y1703S, W1718L and G1770V) have profound functional impact and may be pathogenic variants. By contrast, the other four variants did not alter the function of BRCA1 in the TA assay and should remain classified as VUS. The implementation of combined approaches, such as the one presented here, is therefore of considerable clinical relevance, especially in those cases where co-segregation studies are not possible.

## Supporting Information

Figure S1 Pedigrees of the families with the studied VUS.
(DOC)
