# Supplementary figures and images for: Functional and Structural Analysis of C-Terminal BRCA1 Missense Variants
Source: PLoS One. 2013 Apr 17;8(4):e61302. doi: 10.1371/journal.pone.0061302 (PMC3629201; doi:10.1371/journal.pone.0061302)

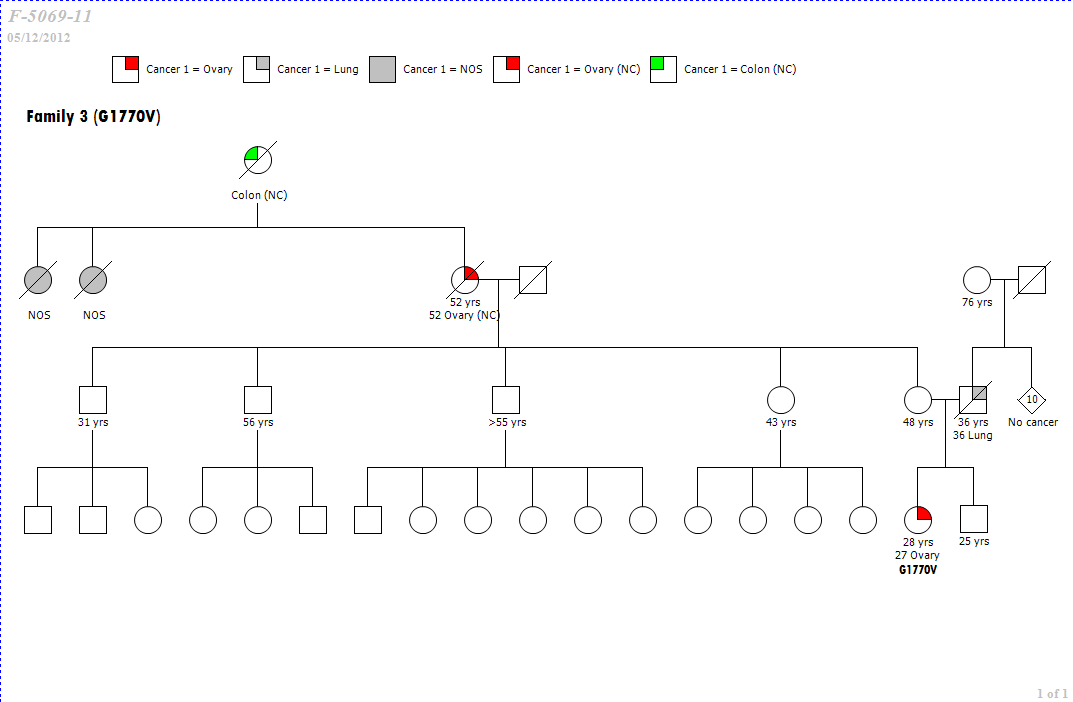


**Family 3 (G1770V)**


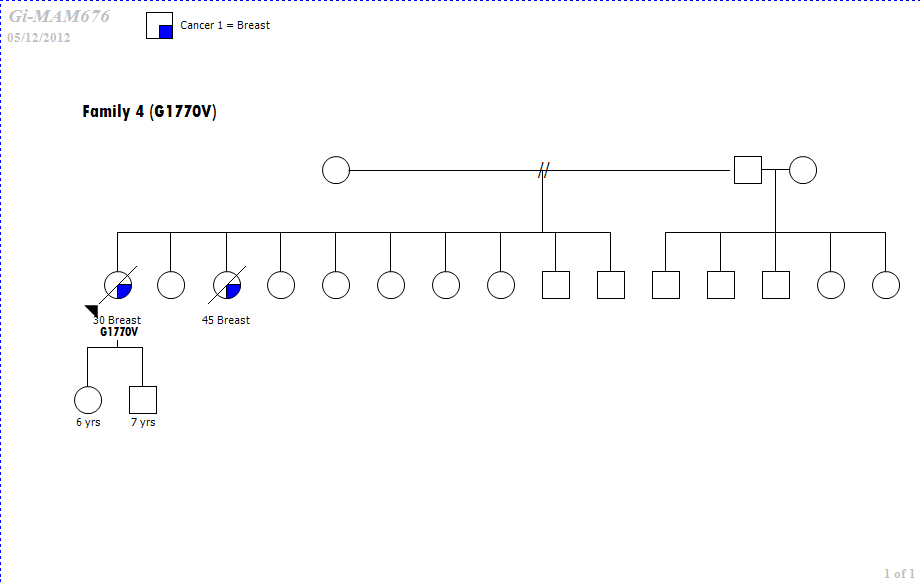


**Family 4 (G1770V)**


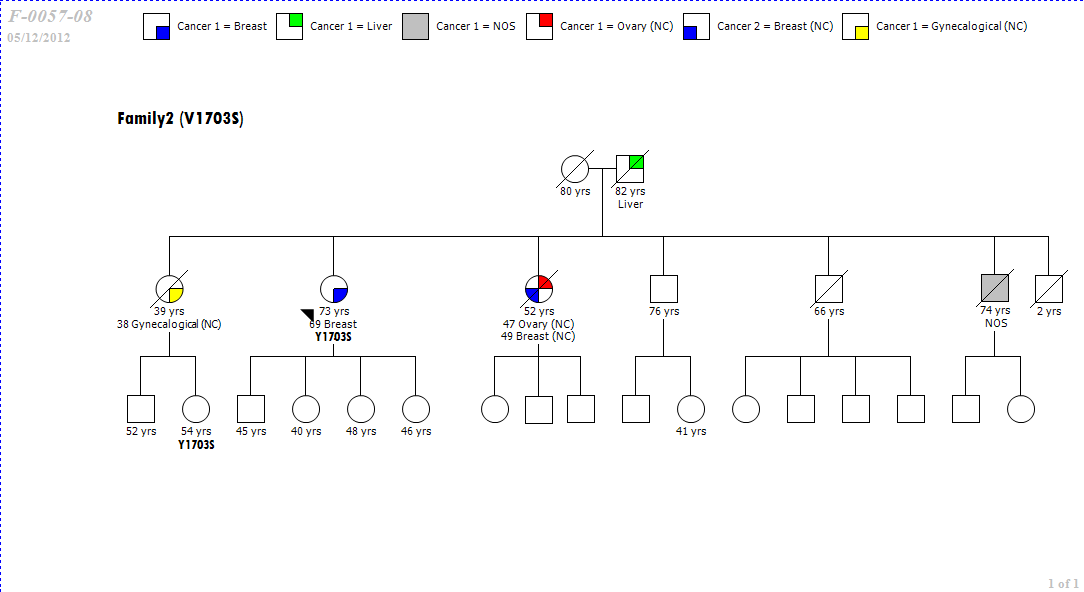


**Family 2 (Y1703S)**


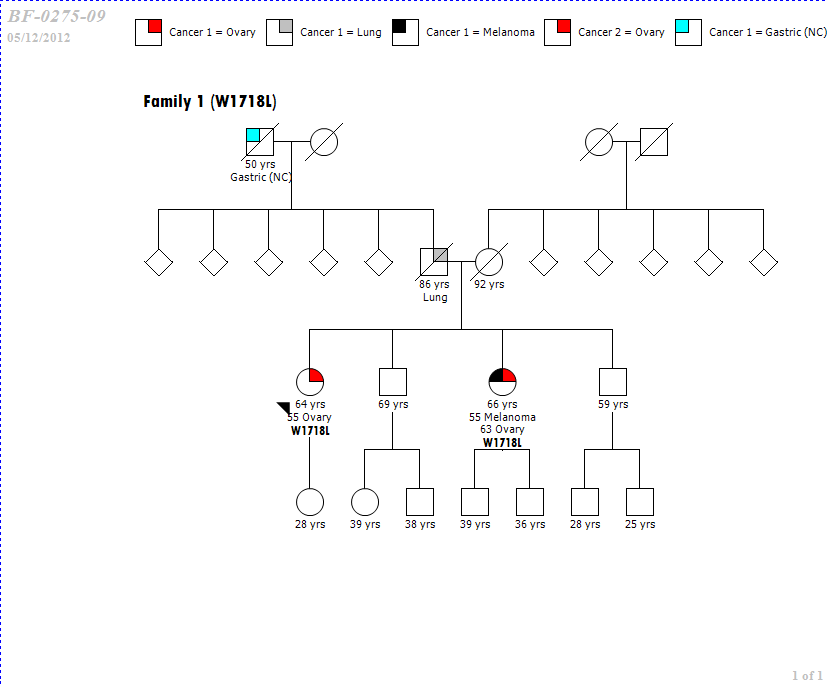


**Family 1 (W1718L)**

**Family 6 (S1409L)**


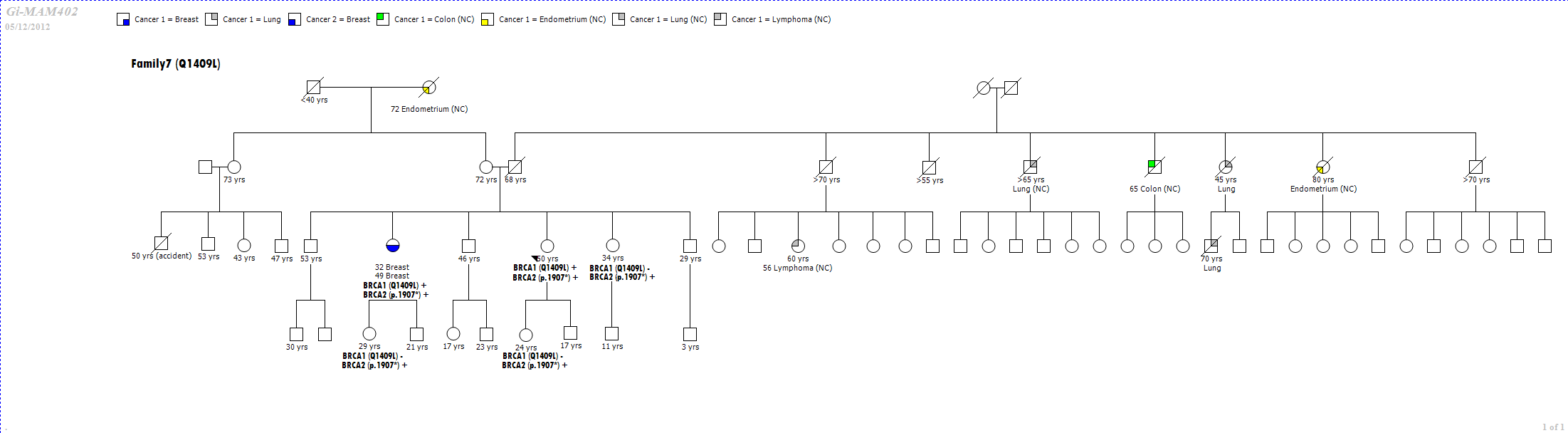

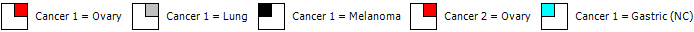

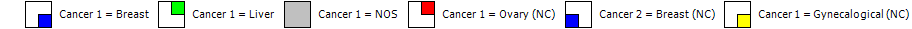

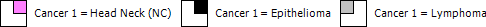

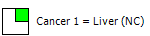

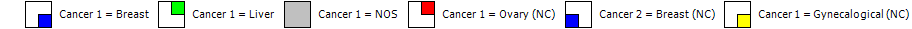

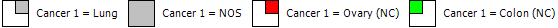

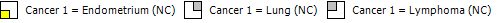

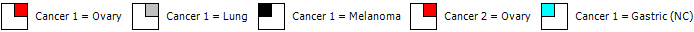

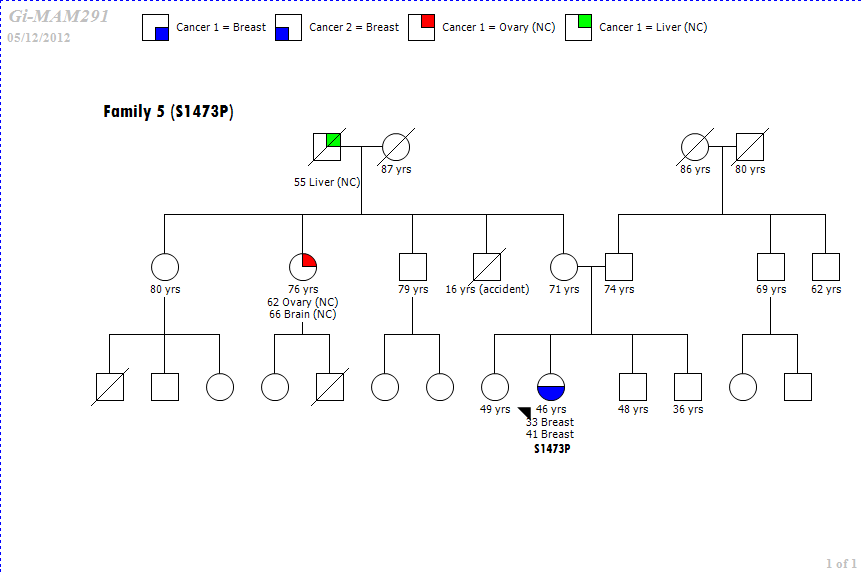


**Family 5 (S1473P)**


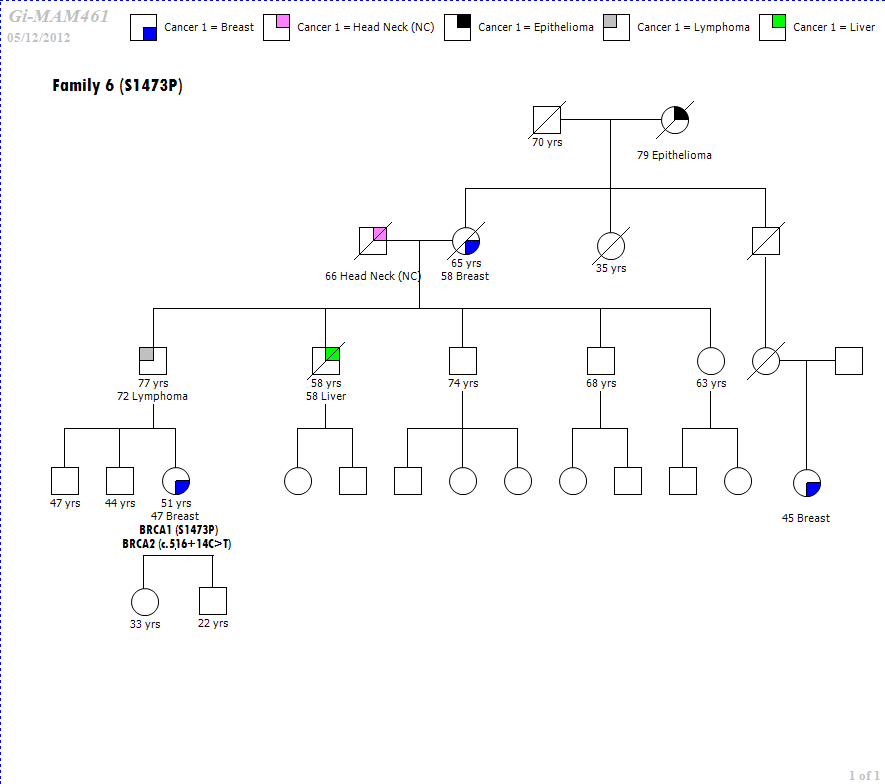


**Family 6 (S1473P)**

Supplement: Figure S1 — Pedigrees of the families with the studied VUS. (DOC) [file pone.0061302.s001.doc]
